# “Prevalence of disordered eating and eating disorders among Norwegian university students before and after the COVID-19 pandemic, 2018 and 2022: The SHoT study.”

**DOI:** 10.1186/s40337-025-01370-3

**Published:** 2025-08-12

**Authors:** Lisa Marie Jacobsen, Gørill Haugan, Gina Dimitropoulos, Amelia Austin, Børge Sivertsen, Tonje Braaten, Ottar Bjerkeset

**Affiliations:** 1 Faculty of Nursing and Health Sciences, Nord University, Levanger, Norway; 2 Department of Health Promotion, Norwegian Institute of Public Health, Bergen, Norway; 3 Faculty of Social Work, University of Calgary, Calgary, Canada; 4 Department of Community Health Sciences, University of Calgary, Calgary, Canada; 5 Department of Research and Innovation, Helse Fonna HF, Haugesund, Norway; 6 Department of Community Medicine, UiT The Arctic University of Norway, Tromsø, Norway

**Keywords:** Eating disorders, Disordered eating, Emerging adults, Post-secondary students, Anorexia nervosa, Bulimia nervosa, Binge eating

## Abstract

**Objective:**

The prevalence of eating disorders (EDs) is increasing, but little is known about their trends among university students, particularly following COVID-19. This study examines prevalence in EDs and disordered eating (DE) symptoms among students in 2018 and 2022, focusing on gender and socio-demographic disparities.

**Methods:**

Data were drawn from the Students’ Health and Well-being Study (SHoT) in 2018 (*n* = 50 054) and 2022 (*n* = 59 544), a large-scale Norwegian survey covering full-time university students aged 18–36. Participants completed self-report measures assessing symptoms of DE (EDS scale) and ED diagnoses, socio-demographic variables, lifestyle, gender identity and financial status. Statistical analyses included logistic regression to assess associations between factors known to be linked to DE and EDs, chi-square tests for group comparisons, and t-tests for continuous variables.

**Results:**

From 2018 to 2022, the prevalence of self-reported EDs increased among females (3.5% in 2018 and 4.5% in 2022) and males (0.4% in 2018 and 0.6% in 2022), while gender-diverse students exhibited the overall highest ED rates (around 10% in both surveys). Anorexia nervosa remained the most common ED among females. Daily/almost daily exercise, financial difficulties, loneliness, and living alone were closely linked to EDs, odds ratios (ORs) ranging from 1.3 to 3.7 in females. Similar patterns were seen for female DE cases. Although numbers were low, financial difficulties and particularly loneliness indicated higher risk for DE and any EDs among males.

**Discussion:**

The findings suggest a relative rise in ED prevalence among students after the COVID-19 pandemic, while DE symptoms and -cases only changed minimally. These findings highlight the need for enhanced awareness and student mental health services, particularly for gender-diverse individuals, and to improve early detection and intervention strategies among those facing financial hardship and loneliness.

**Supplementary Information:**

The online version contains supplementary material available at 10.1186/s40337-025-01370-3.

## Abstract (plain language summary)

This study aimed to identify the prevalence in self-reported eating disorders (EDs) and disordered eating (DE) among Norwegian students before and after the COVID 19 pandemic, and possible associations of DE and ED with gender identity, living conditions, daily/almost daily exercise, financial situation and loneliness in 2018 and 2022. Findings showed that the prevalence of EDs increased among all gender groups. The gender diverse students had an alarmingly high ED prevalence compared to female and male students. The most marked increase was found in anorexia nervosa among female and gender diverse students. Further, our results showed strong associations between loneliness and financial difficulties, and the risk of having DE or an ED diagnosis. These results suggest that there is a need to improve early detection, mental health services and intervention strategies for EDs, particularly for gender diverse students.

**Supplementary Information:**

The online version contains supplementary material available at 10.1186/s40337-025-01370-3.

## Introduction

Eating disorders (EDs) like anorexia nervosa (AN), bulimia nervosa (BN) and binge eating disorder (BED) often emerge in adolescence or young adulthood [1]. The complexity and severity of these disorders are further increased by other psychiatric and medical comorbidities [2], often leading to reduced quality of life and increased health-care costs [3]. The mortality rate is also higher for EDs than most other mental problems and disorders [4]. Genetic, developmental and psychological factors all play a role in the development of EDs [5, 6]. Symptoms of abnormal eating habits fall along a spectrum, and when they do not reach the threshold of an ED diagnosis (i.e., are subclinical) the term disordered eating (DE) is often used [7]. DE represents an “at risk” status [8], can predict ED diagnosis later in life [9], and is associated with long-term health related consequences, particularly psychological distress [10]. Mental health conditions, including EDs, can cause devastating impacts on academic outcomes [11], well-being among students [12], prematurely leaving higher education [13, 14], and suicide among young adults [15].

The overall prevalence of EDs is estimated to be around 2% in Western countries, and considerably higher in females (2.6%) than among males (0.7%) [16]. The global disease burden of EDs increased by 9.4% between 2007 and 2017 according to an Australian rapid review [17]. This trend was also confirmed in studies after COVID-19, of 267.000 college students [18], and 8981 university students [19]. A recent Norwegian study found that new-onset/incident ED cases peaked in 2021, during COVID-19, then declined in 2022 and 2023, yet remained higher than expected based on 10 years of pre-pandemic data [20]. In Norway, the currently estimated prevalence rates of EDs among adolescents (16–19 y/o) are 2.7% for AN, 1.1% for BN, and 1.9% for BED [21]. The ED prevalence among males are comparatively low to females [22], and most studies of EDs in young males to date have been underpowered and therefore yielded limited evidence regarding prevalence estimates and time trends in male student and non-student samples. There is also emerging evidence of markedly higher ED prevalence among gender diverse individuals, yet research remains limited in this group [23, 24].

The literature suggests that loneliness, financial difficulties and excessive exercise are among the modifiable factors associated with ED and DE, as well as their poor outcomes [25–30]. In ED patients, loneliness correlates with increased symptom burden, a marker of severity [31]. Financial difficulties and lower family affluence predicted a worsening in eating attitudes (over time) in a longitudinal study of undergraduate female students [27]. Lastly, about half of individuals with EDs engage in excessive exercise, where patients with AN represent the highest levels [28, 29]. In addition to functioning as an inappropriate compensatory behavior for some individuals, excessive exercise can play a role in the development of an ED as a risk factor. Additionally, increase in physical activity often precedes onset or relapse [32, 33]. Further, pandemic-related factors converged to heighten the risk [34]. This includes disrupted routines, limited support access, social isolation, stress, and adverse effects of social media [35–37]. During the COVID-19 pandemic a considerable increase in underlying risk factors for EDs was seen [36], along with an increased prevalence of EDs and symptoms severity among hospitalized individuals [35, 38]. Among college students, loneliness during the COVID-19 pandemic was associated with a greater risk of EDs. Studies suggest the COVID-19 pandemic impacted on ED-related mental health in the population, particularly affecting younger individuals, females, and those at risk for developing AN [39, 40]. Moreover, after COVID, a cohort study of 350 000 Norwegian adolescents reported a dramatic relative increase in new ED cases among girls (13–16 y/o), reaching 127% in primary care and 96% in specialist care. The number of boys with an ED diagnosis was low, and they were therefore not included in the study [41].

Recently, an online administered diagnostic interview study of 10,000 Norwegian students reported strikingly high rates of mental disorders [42]. Unfortunately, evidence about ED and DE in this population is lacking [18]. There is a need for new (gender specific) estimates of DE and EDs among Norwegian university students, before and after COVID-19, and their associations with socio-demographic factors.

## Aims

We aim to investigate changes in DE and ED estimates, before and after COVID-19 (2018 and 2022), in two National samples of Norwegian full-time university students 18–36 years old. In addition, we will investigate associations of DE and ED with gender identity, living conditions, daily/almost daily exercise, financial situation and loneliness in the same period.

## Methods

### Study design and setting

**Fig. 1:**
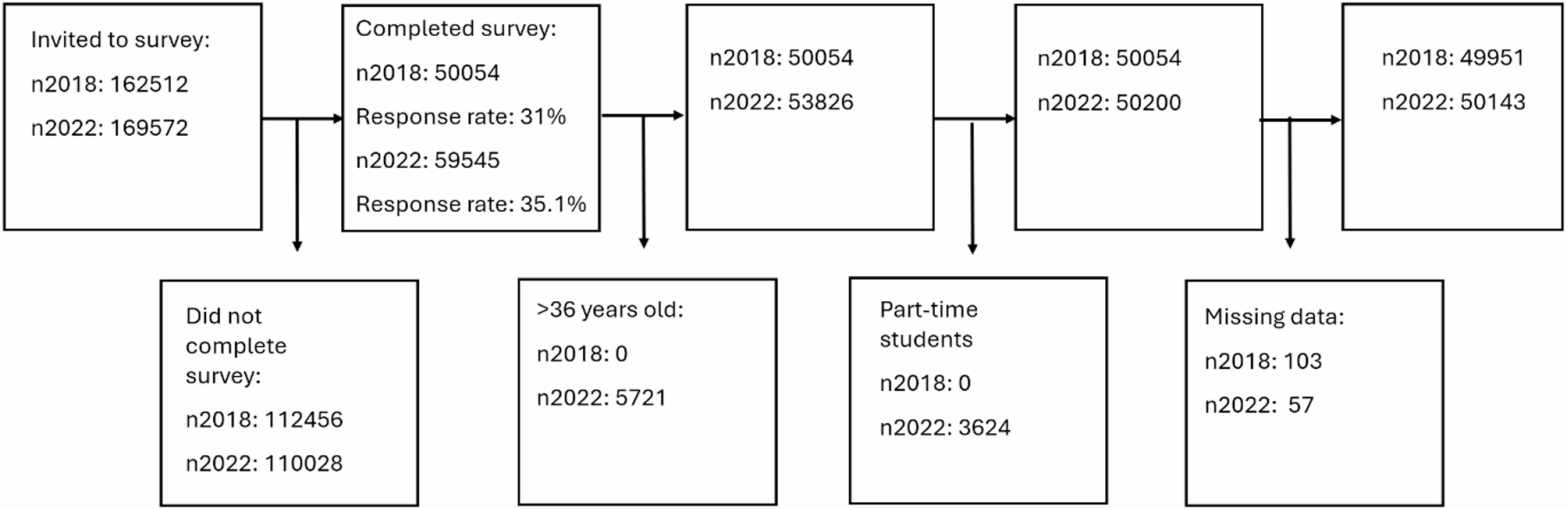
Flow-chart for included students in the SHoT 2018 and 2022 studies, Norway

The Students’ Health and Wellbeing Study (SHoT) is a national serial-entry cohort survey among all Norwegian university full-time students (18–36 years old), carried out in 2018 and 2022. More detailed information about the SHoT survey is published elsewhere [43]. All SHoT surveys were conducted electronically through a web-based platform, including Norwegian students residing in Norway and abroad [42]. SHoT 2018 was carried out between February 6 and April 5, 2018. Out of 162 512 students, 50 054 students completed the survey (response rate 31%), see Fig. 1 [44]. SHoT 2022 took place between February 6 and April 19, 2022, with 59 544 out of 169 572 completed the survey (response rate 35.1%) [45].

#### Outcome variables

**The eating disturbance scale (EDS-5)** is a widely used 5-item self-report questionnaire developed by Rosenvinge and colleagues to measure disturbed eating patterns in adults. We used a version validated on Norwegian students (mean age 25.2) where Cronbach alpha was 0.83 and 0.86, while sensitivity and specificity was 0.90 and 0.88 with respect to DSM-IV EDs in females and males, respectively [46]. The EDS-5 focuses on associated ED behavioural characteristics during the last month, scored on a Likert-scale from 1 to 7 (7 as most severe) [46] : *“Are you satisfied with your eating habits?”*, “*have you eaten to comfort yourself because you were unhappy?”*, “*have you felt guilty about eating?”*, “*have you felt that it was necessary for you to use a strict diet or other eating rituals to control your eating?”*, and lastly, “*have you felt you are too fat?”.* The responses were then dichotomized into 0 (scores 1 through 5) and 1 (scores 6 or 7) for our statistical analyses, to align with the operalization of variables used in other SHoT-studies [45]. Lastly, an EDS cut off > 4.1 was used (DE positive) [47]. In addition to the total score, the EDS-5 items were analyzed and reported for descriptive purposes.

**ED diagnosis** was self-reported, using a comprehensive survey instrument that listed a range of mental and somatic health conditions relevant to the student population. Participants were asked: *”Check the box if you have had any of the following illnesses/conditions (past 12 months)”.* If “mental disorders” were selected, a follow-up list of diagnostic categories were presented, including “Eating disorders” as an option. Participants could then specify subtypes such as “Anorexia”, “Bulimia,” or “Binge eating disorder” [48].

#### Exposure variables

Data on age were extracted from a National Register using the participants’ National 11-digit identity number. The 2018-study asked if the students identified as *“female”*,* “male”*,* “trans”*, or *“others”.* Further, in 2022 “*non-binary”* or *“cross-dressers/others/I don’t know”* were added to the alternatives. In both studies, those who did not identify as either female or male were combined in a gender diverse group.

**BMI** was calculated as weight in kilograms divided by square height in meters. Height and weight were self-reported.

**Daily/almost daily exercise** was assessed using the question: “*On an average week*,* how frequently do you perform physical exercise?”* With possible answers: *“Never”*,* “Less than once a week”*,* “Once a week”*,* “2–3 times per week”* and *“Almost every day”.* The definition of exercise intensity was based on frequency alone, without assessing underlying motivation, psychological distress, or compulsive exercise patterns.

**Financial difficulties** were assessed by asking *“During the last 12 months*,* have you experienced difficulties regarding costs of living?”*, with the possible alternatives: *“never”*,* “rarely”*,* “sometimes”* or *“often”.* These responses were then dichotomized as *“never” and “rarely” to “no”*,* versus “sometimes”* and *“often” to “yes”.*

**Accommodation status** was assessed by following questions: *“do you live alone”? (yes/no).*

**Feeling lonely** was assessed by following questions from “The Three-Item Loneliness Scale (T-ILS)” [49], each rate along a 5-point Likert scale (“*never”*,* “rarely”*,* “sometimes”*,* “often”*,* and “very often*”): “*How often do you feel that you lack companionship?”*,* “How often do you feel left out”*, and “*How often do you feel isolated from others?”.* These responses were dichotomized as *“never”*,* “rarely”* and *“sometimes”* to *“no”*, versus *“often”* and *“very often”* to *“yes”* [50].

### Statistical analysis

Using Stata, version 18 [51], descriptive statistics and multivariable regression analyses were performed. We applied logistic regression models, chi square-test and Fishers exact test for dichotomous outcomes, and independent sample t-test for continuous outcomes.

## Results

Around 50,000 Norwegian full-time students aged 18–36 participated in the SHoT studies in 2018 and 2022. About two thirds of these were females, and one third males. Of these, a group of 115 students (0.2% of total) identified as gender diverse in 2018, this group increased to 340 students (0.7% of total) in 2022. Figure 1 shows the inclusion and participation process.

### Prevalence of disordered eating and eating disorders

From 2018 to 2022, the mean EDS total score increased from 3.5 to 3.6 among female students and from 2.5 to 2.7 among male students, while the proportion of students exceeding the EDS cutoff increased from 33.7 to 36.2% in females and from 11.6 to 12.9% in males. Dissatisfaction with eating habits also increased in the same period among males and females, and more so among male than female students. The number of students that eat to comfort themselves increased in both females and males, yet the proportion in females was almost three times higher than in males (Table 1). Further, both females and males reported feeling more guilt about eating in 2022, compared to 2018, with females almost four times more frequently than male students. In contrast, fewer followed a strict diet in 2022 compared to 2018. Almost 40% of female students and 14% of male students were “feeling too fat” in 2018; these estimates had a minimal decrease in 2022. Overall, gender diverse reported similar, or higher, DE compared to female students. Yet, potential temporal changes did not reach statistical significance in this group, likely due to low numbers.

**Table 1:** Socio-demographic and clinical characteristics of Norwegian students that participated in shot 2018 and 2022

Characteristics	SHoT 2018 (*n* = 49 951)			SHoT 2022 (*n* =50 143)		
	Female (68.9%) (*n* = 34 437)	Male (30.8%) (*n* = 15 399)	Gender diverse (0.2%) (*n* = 115)	Female (65.9%) (*n* = 33 058)	Male (33.4%) (*n* = 16 745)	Gender diverse (0.7%) (*n* = 340)
Age, mean (SD)	23.1 (3.3)	23.5 (3.3)	23.7 (4.2)	23.7 (3.1)	24.1 (3.2)	23.7 (3.2)
**Outcome variables**						
EDS total, mean (SD)	3.5 (1.4)	2.5 (1.2)	3.4 (1.6)	3.6 (1.3)^*^	2.7 (1.1) ^*^	3.5 (1.3)
EDS cut off (%) (N)	33.7 (11 596)	11.6 (1 784)	34.8 (40)	36.2 (11 896)^*^	12.9 (2 138)^*^	31.8 (108)
-dissatisfied with eating habits (%) (N)	13.5% (4 635)	10.9% (1 655)	23.7% (27)	16.4% (5 341) ^*^	16.8% (2 767)^*^	18.7% (63)
-eaten to comfort yourself (%) (N)	5.7% (1 964)	1.7% (264)	6.7% (8)	6.1% (1 988) ^*^	2.2% (357) ^*^	7.1% (24)
-felt guilty about eating (%) (N)	17.9% (6 130)	4.2% (633)	20.9% (24)	20.8% (6 811) ^*^	5.4% (883) ^*^	18.3% (62)
-strict diet to control eating (%) (N)	13.0% (4 458)	5.5% (840)	13.0% (15)	9.9% (3 216) ^*^	4.2% (684) ^*^	12.3% (41)
-felt too fat (%) (N)	36.0% (12 350)	13.7% (2 085)	32.2% (37)	34.8% (11 354) ^*^	13.6% (2 231)	33.5% (112)
Any ED diagnosis (%) (N)	3.5% (1 207)	0.4% (62)	9.6% (11)	4.5% (1 474) ^*^	0.6% (108) ^*^	10.0% (34)
-Anorexia Nervosa (%) (N)	1.9% (667)	0.25% (38)	3.5% (4)	2.6% (860) ^*^	0.3% (47)	5.0% (17)
-Bulimia Nervosa (%) (N)	1.5% (519)	0.1% (10)	2.6% (3)	1.6% (519)	0.2% (27) ^*^	1.8% (6)
-Binge eating disorder (%) (N)	1.0% (347)	0.2% (23)	4.4% (5)	1.4% (470) ^*^	0.3% (44) ^*^	4.1% (14)
**Exposure variables**						
BMI mean (SD)	24.0 (4.5)	24.5(3.9)	25.3 (9.0)	24.1 (4.6) ^*^	24.6 (4.0)	25.3 (6.8)
Daily/almost daily exercise (%) (N)	22.3% (7 643)	26.6% (4 073)	20.2% (23)	15.5% (5 088) ^*^	17.6% (2 903) ^*^	6.9% (23) ^*^
Financial difficulties (%) (N)	32.1% (11 021)	24.4% (3 746)	39.1% (45)	26.5% (8 718) ^*^	21.6% (3 603) ^*^	30.8% (104) ^*^
Living alone (%) (N)	18.7% (6 445)	20.7% (3 189)	28.7% (33)	20.3% (6 708) ^*^	20.2% (3 381)	33.2% (113)
Feeling lonely (%) (N)	10.7% (3 673)	8.9% (1 360)	26.3% (30)	12.4% (4 065) ^*^	10.3% (1 698) ^*^	24.3% (82)

^*^P-value < 0.05 for change, compared to results in SHoT 2018, EDS cut off: >4.1

The prevalence of any self-reported ED diagnosis increased from 2018 to 2022 and was about 8-9-fold higher in female (3.5% and 4.5%) than male (0.4% and 0.6%) students. Yet, numbers were low in the male population (Table 1). However, the highest prevalence in 2018 and 2022 was found in the gender diverse group (9.6% and 10%), but numbers were too low to reach statistical significance for a possible change. More specifically, we observed an increase in all diagnostic categories among females (except BN), and in males (except AN). Notably, the marked increase in any ED diagnosis (total) and AN in female students was explained by increase in those aged ≤ 25, whereas numbers marginally decreased in those ≥ 26 (supplementary S1). Again, among the gender diverse students’ number of cases was too low to establish statistical evidence of change between 2018 and 2022.

Mean BMI essentially stayed the same for all three gender groups from 2018 to 2022 (Table 1). The proportion of students exercising almost every day and having financial difficulties, decreased across all three groups from 2018 to 2022 (Table 1). While the proportion of students living alone showed little change, feeling lonely increased from 2018 to 2022 for both females (10.7% in 2018 and 12.4% in 2022) and males (8.9% in 2018 and 10.3% in 2022). The gender diverse group generally reported greater financial difficulties and more often lived alone, and feeling lonely was two- to threefold more common than in those who identified in the gender binary.

### Associations between exposure variables and disordered eating/eating disorders

For females, we found increased ORs in 2018 and 2022 for any ED in those who exercised daily/almost daily (1.35 in 2018 and 1.76 in 2022), lived alone (1.73 in 2018 and 1.50 in 2022), had financial difficulties (2.20 in 2018 and 2.18 in 2022) and reported feeling lonely (3.69 in 2018 and 3.33 in 2022) (Table 2). Of note, the estimates were robust to further adjustments for each covariate in the model (for fully adjusted model, see supplementary S2a and S2b). The patterns for individual ED subtypes varied, and there was no clear statistical evidence of change over time, except for borderline evidence of an increase in AN.

**Table 2:** : odds ratios (95% CI) for DE and EDs by daily/almost daily exercise, living alone, financial difficulties and feeling lonely in shot 2018 and 2022 (age adjusted)

**Females**	**2018**				
	**DEpos** (*n*: 11 596)	**Any ED** (*n*: 1 207)	**AN** (*n*: 404)	**BN** (*n*: 224)	**BED** (*n*: 167)
Daily/almost daily exercise	0.90 (0.85–0.95)	1.35 (1.19–1.54)	1.51 (1.21–1.87)	1.19 (0.87–1.62)	0.84 (0.57–1.24)
Living alone	1.28 (1.21–1.35)	1.73 (1.52–1.97)	1.93 (1.55–2.40)	1.37 (1.00-1.88)	2.31 (1.67–3.19)
Financial difficulties	1.99 (1.90–2.09)	2.20 (1.95–2.47)	1.83 (1.50–2.24)	2.21 (1.69–2.88)	2.70 (1.98–3.69)
Feeling lonely	3.14 (2.93–3.75)	3.69 (3.23–4.20)	3.60 (2.89–4.50)	2.96 (2.17–4.04)	4.70 (3.40–6.52)
	**2022**				
	**DEpos** (*n*: 11 896)	**Any ED** (*n*: 1 474)	**AN** (*n*: 546)	**BN** (*n*: 212)	**BED** (*n*: 230)
Daily/almost daily exercise	0.93 (0.87–0.99)	1.76 (1.55–1.99)	2.24 (1.85–2.71)	1.68 (1.22–2.32)	1.23 (0.88–1.74)
Living alone	1.26 (1.19–1.33)	1.50 (1.33–1.69)	1.53 (1.27–1.86)	1.21 (0.88–1.67)	1.47 (1.09–1.97)
Financial difficulties	1.98 (1.84–2.09)	2.18 (1.96–2.43)	1.73 (1.45–2.07)	2.62 (2.00-3.45)	2.71 (2.09–3.52)
Feeling lonely	2.66 (2.49–2.85)	3.33 (2.96–3.74)	3.21 (2.75-4.00)	2.42 (1.77–3.33)	5.01 (3.83–6.54)

DEpos: disordered eating positive, any ED: any eating disorder, AN: anorexia nervosa, BN: bulimia nervosa, BED: binge eating disorder. Reference groups: DE and ED negative students, respectively

**Table 3:** : odds ratios (95% CI) for DE and EDs by daily/almost daily exercise, living alone, financial difficulties and feeling lonely in shot 2018 and 2022 (age adjusted)

**Males**	**2018**				
	**DEpos** (*n*: 1 784)	**Any ED** (*n*: 62)	**AN** (*n*: 26)	**BN** (*n*: 1)	**BED** (*n*: 15)
Daily/almost daily exercise	0.68 (0.60–0.77)	1.15 (0.66-2.00)	1.22 (0.53–2.81)	Not applicable	1.58 (0.53–4.67)
Living alone	1.66 (1.49–1.86)	1.65 (0.96–2.85)	2.42 (1.09–5.36)	Not applicable	0.48 (0.10–2.17)
Financial difficulties	2.05 (1.84–2.27)	2.32 (1.39–3.86)	2.76 (1.26–6.03)	Not applicable	3.81 (1.34–10.83)
Feeling lonely	4.28 (3.76–4.87)	8.42 (5.08–13.97)	12.42 (5.72–26.98)	Not applicable	5.88 (2.06–16.72)
	**2022**				
	**DEpos** (*n*: 2 138)	**Any ED** (*n*: 108)	**AN** (*n*: 29)	**BN** (*n*: 12)	**BED** (*n*: 28)
Daily/almost daily exercise	0.74 (0.65–0.84)	1.07 (0.65–1.74)	1.19 (0.48–2.94)	0.95 (0.20–4.38)	1.34 (0.54–3.32)
Living alone	1.34 (1.20–1.49)	2.42 (1.63–3.59)	2.21 (1.01–4.78)	3.87 (1.23–12.11)	1.67 (0.75–3.74)
Financial difficulties	2.25 (2.05–2.50)	2.75 (1.86–4.06)	3.97 (1.88–8.40)	1.15 (0.30–4.32)	2.10 (0.97–4.52)
Feeling lonely	3.36 (2.99–3.78)	4.81 (3.22–7.17)	2.91 (1.24–6.83)	4.34 (1.30-14.49)	6.22 (2.92–13.23)

DEpos: disordered eating positive, any ED: any eating disorder, AN: anorexia nervosa, BN: bulimia nervosa, BED: binge eating disorder. Reference groups: DE and ED negative students, respectively

(Table 2). For females that reported feeling lonely, the results showed an increased risk of DE (3.14 in 2018 and 2.66 in 2022), any ED (3.69 in 2018 and 3.33 in 2022), and all subtypes in both 2018 and 2022, with the BED in 2022 showing the highest risk (5.01).

For males (Table 3), increased risks for any ED and AN remained elevated in both 2018 and 2022 in students with financial difficulties, were any ED (2.32 in 2018 and 2.75 in 2022) and AN (2.76 in 2018 and 3.97 in 2022) were most prominent. For males reporting feeling lonely, the results showed an elevated risk of DE (4.28 in 2018 and 3.36 in 2022), any ED (8.42 in 2018 and 4.81 in 2022), and all subtypes in both 2018 and 2022, with the AN risk in 2018 being the most increased (12.42).

## Discussion

The mental health of university students is an important global concern; recently mental health issues have increased markedly. Considering the long-term benefits of early identification and prompt management of mental health disorders, there is a need for understanding the current prevalence of EDs before and after the COVID-19 pandemic. In two large national samples of Norwegian full-time university students from 2018 to 2022 (one year after the major COVID-19 restrictions were lifted in Norway), we were able to show an increase in self-reported EDs in both male and female students. ED occurrence was 8-9-fold higher in females than males yet estimates in the gender diverse group were approximately 2–3 fold higher than those of the female group. In contrast, symptom level of DE and DE cases only showed a minimal to small increase in all three gender groups between 2018 and 2022. Overall, daily/almost daily exercise, living alone, financial difficulties, and particularly loneliness were significantly associated with DE and EDs. In sum, our findings are consistent with the existing literature, showing an increased prevalence of EDs after the pandemic [19, 35, 41, 52]. In addition, we found an alarmingly high prevalence of EDs and ED-symptomatology among gender diverse students, in keeping with recent evidence [23, 24].

We found a marked relative increase in EDs prevalence after the pandemic, which might relate to several pandemic-related stressors. Findings from a qualitative study of 129 adults with symptoms compatible to EDs, showed that social isolation gave ED patients the possibility to cover up ED symptomatology [53]. Furthermore, McCombie et al. 2020 found that pandemic experiences, including isolation, routine disruption, and media messages worsened ED cognitions and behaviors, and increased anxiety and depression symptoms in individuals with lifetime EDs [54]. Accordingly, our findings seems to fit recent results from longitudinal Norwegian health registry studies reporting an increase in ED prevalence after the pandemic [20]. The increase in self-reported ED diagnoses, compared to smaller changes in mean EDS-5 scores and prevalence of DE cases and may reflect differences in measurement: the ED diagnosis item captures 12-months prevalence, whereas the EDS-5 assesses current symptoms. Thus, students’ report of an ED diagnosis in 2022 might reflect diagnostic levels during COVID, while DE symptom levels had already declined at the time of the survey. Additionally, growing awareness and improved access to diagnostic services in recent years may have contributed to higher rates of formal diagnoses, even if current subclinical symptoms in the population remain stable. The EDS-5 may also not fully capture all ED presentations, which could further explain the discrepancy. Nevertheless, the prevalence of AN among females in our study (AN = 2.6%) is very similar to a previous Norwegian study from 2023 (AN = 2.7%) among female adolescents using self-reported measures [21]. Based on the design of this study, we cannot assume any causal relationship between potential risk factors and EDs, and future cohort studies applying longitudinal designs should assess this. Schools and universities in Norway were closed during the first COVID-19 wave, and gradually re-opened [55]. During later waves, they remained open. Although the surge in EDs during the COVID-19 pandemic occurred worldwide, lockdowns policies were local, and post-pandemic ED trends can differ in different countries.

The strikingly high ED and DE prevalences in the gender diverse students suggest that this group is especially vulnerable and deserves further attention, both from clinicians and researchers. These results are similar to a systematic review [56], a study of 660 adolescences [57], and a study on 286,720 U.S. college students [58], finding an increased risk of EDs and rate of lifetime ED diagnosis among gender diverse individuals. Results from a systematic review by Rasmussen at al. 2023 that included 14 European and US studies indicated that gender diverse individuals, both students and other adults, present with higher levels of ED symptomatology relative to cisgender individuals, especially cisgender men [23]. Gender diverse students might also experience more social stress and stigma, like harassment and discrimination compared to their cisgender peers [59]. Experience transphobic stigma was linked to higher levels of DE behaviors [60]. In keeping with our results, a SHoT-study published before the Covid-19 pandemic in 2020 on the same cohort of gender diverse students reported higher prevalence of psychosocial distress, loneliness, suicidal ideation, suicidal behavior, and self-harm than in binary female and male students [61]. Ålgars et al. suggest that the most frequent reason for EDs among gender diverse individuals is the drive to achieve thinness as a way to suppress characteristics of the sex assigned at birth, or to enhance characteristics of the gender identity [62], using eating and diet habits to modify sex characteristics and/or gender expression [63]. Moreover, ED psychopathology in these two groups is seen to associate with factors like stigma, discrimination, and internalized transphobia, all of which may contribute to the development of ED behaviors [60, 64, 65]. ED treatment was traditionally created for cisgender individuals [66], with measurements developed and validated on females [67]. Further, gender affirming health care services are lacking [66], and this could be possible explanations for the worrisome trends reported by gender diverse students.

In our study, students that exercised daily/almost daily, lived alone, reported financial difficulties, and feeling lonely, displayed elevated risk of presenting with EDs. The same was seen for DE, except that daily exercise was associated with lower ORs of DE. Notably, living alone and feeling lonely did not exert the same influence: although both were linked to higher ED risk, loneliness showed the largest effect sizes across outcomes. This underscores the importance of subjective social connectedness, beyond physical living arrangements, in understanding ED vulnerability. A longitudinal exploratory study of 1568 American emerging adults found an association between female and gender diverse individuals, and financial difficulties, living situation, and risk for DE [26]. Further, greater financial difficulties predict a worsening in eating attitudes over time in female student [27]. Tavolacci et al. (2015) also found an increased risk of EDs among students with the social determinants; stress, depression and cyber-addiction [68]. Moreover, loneliness during the COVID-19 pandemic is seen to associate with a 10% greater risk of ED symptomatology among college students, and even more pronounced in males compared to females [25]. We found increased ORs among female university students exercising almost daily and any ED and AN. This is comparable to previous research, showing that excessive exercise is a commonly observed behavior in EDs, especially in AN [29]. A systematic review claims that there is a reasonably consistent association between concern early in life with weight and shape, and negative affect on later excessive exercise [69]. Among students experiencing stress, excessive exercise behavior could be seen as a coping mechanism, combined with social and cultural pressures of body image.

Moreover, we found increased ORs of both female and male students with BED and financial difficulties. People with BED might have an extraordinarily high food budget, and this finding could potentially be seen in the context of high and rapidly increasing Norwegian food prices. The association between BED and financial difficulties is also consistent with findings from Mikhail et al. 2023 claiming that university students who experienced greater financial hardship had significantly greater dimensional binge eating symptoms [70]. Disseminating ED interventions (either group or internet based) in university settings could be a potential way to reduce the incidence of subthreshold, and potentially threshold EDs [71].

**Strengths and limitations**.

The SHoT study has some strengths that should be mentioned. First, and most importantly, the large sample size including about 50,000 students in both waves, 2018 and 2022, cover all universities in Norway. Second, a rich data set makes it possible to study a wide range of health issues, and their social demographic and lifestyle correlates. Third, the possibility to study the development of EDs and DE among cis- and the quickly emerging gender diverse groups in the same study setting makes for a reliable and novel approach.

Some limitations should also be considered when interpreting our findings. First, this was a convenience sample, and selection bias resulted in more women than men responding to the survey, combined with a relatively low response rate (31% in 2018 and 35.1% in 2022). However, it should be mentioned that the attendance rate and gender rates were quite stable across universities and across timepoints, and we therefore argue that estimates are suited to estimate relative change in prevalence between 2018 and 2022. Our data were all self-report, and while EDS-5 addresses DE symptoms last 30 days, ED diagnoses were reported of the last 12-months. This difference should be taken into consideration while interpreting the results. It should be noted that the students included in our study were up to 36 years old, and a wider age range than in other studies [19]. Further, our operationalization of intensity of exercise (daily/almost daily) was based solely on frequency, which is a limitation. The measure did not capture whether exercise was driven, compulsive, or used as a compensatory behaviour, factors that are highly relevant in the context of EDs. A more comprehensive assessment would have included cognitive and motivational components. Finally, the male and gender diverse group were relatively small, and findings related to these groups should be interpreted with caution.

## Conclusion

The current study points to an increase in prevalence of self-reported EDs among Norwegian university students from 2018 to 2022 and further identified strong associations of DE and ED with financial situation, and, particularly, loneliness and gender diversity. In conclusion, these findings underscore the need for improved information, support and ED programming on university campuses and in student unions. Finally, there is a need for better understanding of students with DE and EDs, particularly among the gender diverse, and the importance of low-threshold and effective treatments.

## Supplementary Information

Below is the link to the electronic supplementary material.

Supplementary Material 1

Supplementary Material 2

## Data Availability

The data collected and analyzed are not available due to ethical considerations observed to maintain the participants’ anonymity in the SHoT survey.

## Abbreviations

DE: Disordered eating
EDs: eating disorders
AN: Anorexia nervosa
BN: Bulimia nervosa
BED: Binge eating disorder
OR: Odds ratio
SHoT: The Students’ Health and Wellbeing Study
