# Correction: “Prevalence of disordered eating and eating disorders among Norwegian university students before and after the COVID-19 pandemic, 2018 and 2022: the SHoT study.”

**DOI:** 10.1186/s40337-025-01389-6

**Published:** 2025-08-25

**Authors:** Lisa Marie Jacobsen, Gørill Haugan, Gina Dimitropoulos, Amelia Austin, Børge Sivertsen, Tonje Braaten, Ottar Bjerkeset

**Affiliations:** 1 Faculty of Nursing and Health Sciences, Nord University, Levanger, Norway; 2 Department of Health Promotion, Norwegian Institute of Public Health, Bergen, Norway; 3 Faculty of Social Work, University of Calgary, Calgary, Canada; 4 Department of Community Health Sciences, University of Calgary, Calgary, Canada; 5 Department of Research and Innovation, Helse Fonna HF, Haugesund, Norway; 6 Department of Community Medicine, UiT The Arctic University of Norway, Tromsø, Norway

**Correction to: Jacobsen et al. Journal of Eating Disorders (2025) 13:173**

10.1186/s40337-025-01370-3

Following the publication of the original article, the authors reported that there was a typo in the title of the article. The year 2022 was erroneously written as 202.

The correct title should be: “**Prevalence of disordered eating and eating disorders among Norwegian university students before and after the COVID-19 pandemic**,** 2018 and 2022: The SHoT study**.”

Further, there was a typo in the method section, page 6 line 155 and line 160:

The likert-scale should be 1–7, not 0–7.

The correct text should be: “The EDS-5 focuses on associated ED behavioural characteristics during the last month, scored on a Likert-scale from 1 to 7 (7 as most severe) (46) : *“Are you satisfied with your eating habits?”*, “*have you eaten to comfort yourself because you were unhappy?”*, “*have you felt guilty about eating?”*, “*have you felt that it was necessary for you to use a strict diet or other eating rituals to control your eating?”*, and lastly, “*have you felt you are too fat?”.* The responses were then dichotomized into 0 (scores 1 through 5) and 1 (scores 6 or 7) for our statistical analyses, to align with the operalization of variables used in other SHoT-studies (45).”

The original article has been updated accordingly.

