# Supplementary material for: “Prevalence of disordered eating and eating disorders among Norwegian university students before and after the COVID-19 pandemic, 2018 and 2022: The SHoT study.”
Source: J Eat Disord. 2025 Aug 12;13:173. doi: 10.1186/s40337-025-01370-3 (PMC12341137; doi:10.1186/s40337-025-01370-3)
Supplement: Supplementary file 1 — Supplementary Material 1 [file 40337_2025_1370_MOESM1_ESM.docx]

**Supplementary Table S1a:** Prevalence of DE and ED for female DE- and ED-cases in SHoT 2018 and 2022, stratifies by age groups.

| **Females** | **2018** | | | **2022** | | |
| --- | --- | --- | --- | --- | --- | --- |
|  | **Age 18-22** | **Age 23-25** | **Age 26-36** | **Age 18-22** | **Age 23-25** | **Age 26-36** |
| EDS total mean, (SD) | 3.44 (1.4) | 3.48 (1.4) | 3.58 (1.5) | 3.57 (1.3) | 3.57 (1.3) | 3.62 (1.4) |
| -dissatisfied with eating habits, mean, (SD) | 3.69 (1.6) | 3.71 (1.6) | 3.77 (1.6) | 4.03 (1.5) | 3.99 (1.5) | 4.02 (1.5) |
| -eaten to comfort yourself, mean, (SD) | 2.90 (1.5) | 2.99 (1.5) | 3.16 (1.6) | 3.12 (1.5) | 3.11 (1.6) | 3.23 (1.7) |
| -felt guilty about eating, mean, (SD) | 3.56 (1.9) | 3.57 (1.9) | 3.61 (1.9) | 3.84 (1.9) | 3.76 (1.9) | 3.66 (1.9) |
| -strict diet to control eating, mean, (SD) | 2.80 (1.9) | 2.83 (1.9) | 2.93 (2.0) | 2.60 (1.9) | 2.64 (1.8) | 2.71 (1.9) |
| -felt too fat, mean, (SD) | 4.23 (2.1) | 4.31 (2.1) | 4.44 (2.1) | 4.27 (2.1) | 4.33 (2.1) | 4.49 (2.1) |
| ED diagnosis, (%) | 3.49 | 3.40 | 3.76 | 4.76 | 4.46 | 3.59 |
| Anorexia Nervosa, (%) | 2.08 | 1.85 | 1.71 | 2.96 | 2.61 | 1.56 |
| Bulimia Nervosa, (%) | 1.4 | 1.54 | 1.78 | 1.61 | 1.56 | 1.47 |
| Bige eating disorder, (%) | 0.8 | 1.16 | 1.37 | 1.41 | 1.34 | 1.59 |

**Supplementary Table S1b:** Prevalence of DE and ED for female DE- and ED-cases in SHoT 2018 and 2022, stratifies by age groups.

| **Males** | **2018** | | | **2022** | | |
| --- | --- | --- | --- | --- | --- | --- |
|  | **Age 18-22** | **Age 23-25** | **Age 26-36** | **Age 18-22** | **Age 23-25** | **Age 26-36** |
| EDS total mean, (SD) | 2.40 (1.1) | 2.50 (1.1) | 2.71 (1.3) | 2.58 (1.1) | 2.64 (1.1) | 2.86 (1.2) |
| -dissatisfied with eating habits, mean, (SD) | 3.34 (1.6) | 3.36 (1.6) | 3.45 (1.6) | 3.92 (1.6) | 3.90 (1.6) | 3.99 (1.5) |
| -eaten to comfort yourself, mean, (SD) | 1.89 (1.2) | 1.99 (1.3) | 2.21 (1.4) | 2.08 (1.4) | 2.12 (1.4) | 2.39 (1.5) |
| -felt guilty about eating, mean, (SD) | 2.18 (1.5) | 2.25 (1.5) | 2.41 (1.6) | 2.39 (1.6) | 2.43 (1.6) | 2.55 (1.7) |
| -strict diet to control eating, mean, (SD) | 1.97 (1.5) | 2.06 (1.6) | 2.25 (1.7) | 1.83 (1.5) | 1.89 (1.5) | 2.08 (1.6) |
| -felt too fat, mean, (SD) | 2.62 (1.9) | 2.84 (1.9) | 3.21 (2.0) | 2.65 (1.9) | 2.87 (2.0) | 3.30 (2.1) |
| ED diagnosis, (%) | 0.43 | 0.27 | 0.60 | 0.68 | 0.55 | 0.72 |
| Anorexia Nervosa, (%) | 0.30 | 0.15 | 0.31 | 0.31 | 0.28 | 0.22 |
| Bulimia Nervosa, (%) | 0.10 | 0.04 | 0.03 | 0.18 | 0.12 | 0.19 |
| Bige eating disorder, (%) | 0.13 | 0.10 | 0.28 | 0.19 | 0.28 | 0.39 |

**Supplementary Table S1c:** Prevalence of DE and ED for female DE- and ED-cases in SHoT 2018 and 2022, stratifies by age groups.

| **Gender diverse** | **2018** | | | **2022** | | |
| --- | --- | --- | --- | --- | --- | --- |
|  | **Age 18-22** | **Age 23-25** | **Age 26-36** | **Age 18-22** | **Age 23-25** | **Age 26-36** |
| EDS total mean, (SD) | 3.54 (1.5) | 3.27 (1.6) | 3.17 (1.6) | 3.53 (1.4) | 3.49 (1.3) | 3.53 (1.4) |
| -dissatisfied with eating habits, mean, (SD) | 4.44 (1.6) | 4.58 (1.8) | 3.55 (1.8) | 4.05 (1.7) | 4.10 (1.4) | 3.58 (1.3) |
| -eaten to comfort yourself, mean, (SD) | 3.00 (1.7) | 2.25 (1.6) | 3.44 (2.0) | 3.34 (1.7) | 3.36 (1.5) | 3.13 (1.6) |
| -felt guilty about eating, mean, (SD) | 3.68 (2.1) | 3.16 (2.2) | 2.85 (2.1) | 3.63 (2.0) | 3.49 (1.9) | 3.55 (1.9) |
| -strict diet to control eating, mean, (SD) | 2.88 (1.9) | 2.45 (2.0) | 2.48 (2.0) | 2.57 (2.0) | 2.45 (1.8) | 2.86 (2.2) |
| -felt too fat, mean, (SD) | 3.74 (2.5) | 3.58 (2.4) | 3.51 (2.6) | 4.06 (2.2) | 3.99 (2.3) | 4.49 (2.1) |
| ED diagnosis, mean, (SD) | 14.0 | 9.68 | 3.7 | 11.18 | 7.56 | 11.67 |
| Anorexia Nervosa, mean, (SD) | 6.0 | 0 | 3.7 | 5.59 | 3.36 | 6.67 |
| Bulimia Nervosa, mean, (SD) | 4.0 | 0 | 3.7 | 1.86 | 1.68 | 1.67 |
| Bige eating disorder, mean, (SD) | 6.0 | 3.23 | 3.70 | 4.35 | 1.68 | 8.33 |
