# Supplementary material for: “Prevalence of disordered eating and eating disorders among Norwegian university students before and after the COVID-19 pandemic, 2018 and 2022: The SHoT study.”
Source: J Eat Disord. 2025 Aug 12;13:173. doi: 10.1186/s40337-025-01370-3 (PMC12341137; doi:10.1186/s40337-025-01370-3)
Supplement: Supplementary file 2 — Supplementary Material 2 [file 40337_2025_1370_MOESM2_ESM.docx]

| **Females** | 2018 | | | | |
| --- | --- | --- | --- | --- | --- |
|  | DEpos (n: 11 596) | Any ED (n: 1 207) | AN (n: 404) | BN (n: 224) | BED (n: 167) |
| Exercise almost daily | 0.93 (0.88-0.99) | 1.42 (1.24-1.62) | 1.58 (1.27-1.97) | 1.24 (0.90-1.68) | 0.91 (0.61-1.35) |
| Living alone | 1.16 (1.10-1.24) | 1.52 (1.33-1.74) | 1.74 (1.40-2.17) | 1.24 (0.90-1.70) | 1.91 (1.37-2.66) |
| Financial difficulties | 1.86 (1.77-1.95) | 1.94 (1.72-2.19) | 1.64 (1.33-2.01) | 2.01 (1.53-2.65) | 2.13 (1.55-2.94) |
| Feeling lonely | 2.88 (2.68-3.09) | 3.29 (2.88-3.76) | 3.29 (2.63-4.13) | 2.65 (1.93-3.63) | 3.75 (2.68-5.24) |
|  | 2022 | | | | |
|  | DEpos (n: 11 896) | Any ED (n: 1 474) | AN (n: 546) | BN (n: 212) | BED (n: 230) |
| Exercise almost daily | 0.95 (0.89-1.01) | 1.84 (1.62-2.08) | 2.30 (1.90-2.79) | 1.77 (1.28-2.44) | 1.29 (0.91-1.81) |
| Living alone | 1.15 (1.09-1.22) | 1.31 (1.16-1.48) | 1.35 (1.11-1.64) | 1.09 (0.78-1.50) | 1.20 (0.89-1.61) |
| Financial difficulties | 1.85 (1.76-1.95) | 1.94 (1.74-2.17) | 1.52 (1.27-1.82) | 2.53 (1.92-3.33) | 2.23 (1.70-2.91) |
| Feeling lonely | 2.43 (2.27-2.61) | 3.01 (2.67-3.40) | 3.17 (2.61-3.83) | 2.19 (1.58-3.02) | 4.33 (3.30-5.70) |

**Supplementary Table S2a.** Odds ratios (95% CI) for excessive exercise, living alone, having financial difficulties and feeling lonely in female DE- and ED-cases in SHoT 2018 and 2022. Reference groups: DE and ED negative students, respectively.

*All covariates adjusted for each other

DEpos: disordered eating positive, any ED: any eating disorder, AN: anorexia nervosa, BN: bulimia nervosa, BED: binge eating disorder.

**Supplementary Table S2b**. Odds ratios (95% CI) for excessive exercise, living alone, having financial difficulties and feeling lonely in male DE- and ED-cases in SHoT 2018 and 2022. Reference groups: DE and ED negative students, respectively.

| **Males** | 2018 | | | | |
| --- | --- | --- | --- | --- | --- |
|  | DEpos (n: 1 784) | Any ED (n: 62) | AN (n: 26) | BN (n: 1) | BED (n: 15) |
| Exercise almost daily | 0.72 (0.64-0.82) | 1.33 (0.76-2.32) | 1.46 (0.63-3.40) | Not applicable | 1.84 (0.61-5.48) |
| Living alone | 1.46 (1.30-1.64) | 1.25 (0.72-2.19) | 1.77 (0.79-3.98) | Not applicable | 0.34 (0.07-1.58) |
| Financial difficulties | 1.87 (1.68-2.09) | 1.88 (1.12-3.17) | 2.15 (0.97-4.77) | Not applicable | 3.26 (1.12-9.44) |
| Feeling lonely | 3.76 (3.29-4.29) | 7.58 (4.49-12.78) | 10.51 (4.72-23.42) | Not applicable | 5.78 (1.97-16.97-) |
|  | 2022 | | | | |
|  | DEpos (n: 2 138) | Any ED (n: 108) | AN (n: 29) | BN (n: 12) | BED (n: 28) |
| Exercise almost daily | 0.78 (0.68-0.89) | 1.15 (0.69-1.89) | 1.13 (0.42-2.98) | 1.03 (0.22-4.75) | 1.51 (0.60-3.74) |
| Living alone | 1.16 (1.03-1.29) | 1.96 (1.31-2.94) | 1.96 (0.89-4.31) | 3.29 (1.03-10.51) | 1.26 (0.55-2.87) |
| Financial difficulties | 2.04 (1.84-2.26) | 2.24 (1.50-3.33) | 3.47 (1.62-7.44) | 0.91 (0.23-48) | 1.66 (0.76-3.63) |
| Feeling lonely | 3.00 (2.66-3.38) | 3.76 (2.48-5.69) | 2.12 (0.87-5.13) | 3.48 (1.00-12.10) | 5.48 (2.51-11.96) |

*All covariates adjusted for each other

DEpos: disordered eating positive, any ED: any eating disorder, AN: anorexia nervosa, BN: bulimia nervosa, BED: binge eating disorder.
